# Paget’s Breast Disease: A Case Report and Review of the Literature

**DOI:** 10.3389/fsurg.2017.00051

**Published:** 2017-10-23

**Authors:** S. Dubar, M. Boukrid, Jean Bouquet de Joliniere, L. Guillou, Quoc Duy Vo, A. Major, N. Ben Ali, F. Khomsi, A. Feki

**Affiliations:** ^1^Department of Gynecology-Obstetric, Cantonal Hospital of Fribourg, Fribourg, Switzerland

**Keywords:** female breast cancer, breast Paget’s disease, biopsy, imagery, surgery, sentinel lymph node biopsy

## Abstract

Paget’s disease of the breast is a rare cancer. This typical clinical case illustrates the different epidemiological, clinical, histological, therapeutic, and evolving aspects of the disease. We report a case of Paget’s disease in a 43-year-old woman who presented eczema of the nipple. Mammography and ultrasounds did reveal a lesion *in situ*. The patient was scheduled for mastectomy and sentinel node biopsy. She had chosen a radical bilateral surgery. The histological diagnosis was Paget’s disease of the breast with a carcinoma *in situ*. There was no metastasis in either of the sentinel nodes. Paget’s disease must be considered with the presence of a persistent eczematous involvement of the nipple, which does not respond to local treatment. Ultrasounds, mammography, and magnetic resonance imaging can allow searching an underlying cancer and guiding the surgical management. There is no evidence at this time that one of the two surgical techniques (conservative or mastectomy) would improve survival. The prognosis depends on the presence of a palpable mass and the invasiveness of the cancer.

## Background

Paget’s disease of the breast is a rare histological breast cancer, representing 1–3% of female breast cancers. It appears as an isolated affection on 1.4–13% of cases and is associated with an *in situ* or invasive glandular carcinoma on 90–100% of cases. *In situ* histology is found on 1/3 cases. The average age of onset of disease is 56 years old. The surgical treatment of Paget’s disease is controversial (radical or conservative). The purpose of this article is to discuss the epidemiological, clinical, histological, therapeutic, and prognostic aspects of the Paget’s disease.

## Case Presentation

**Figure 1:**
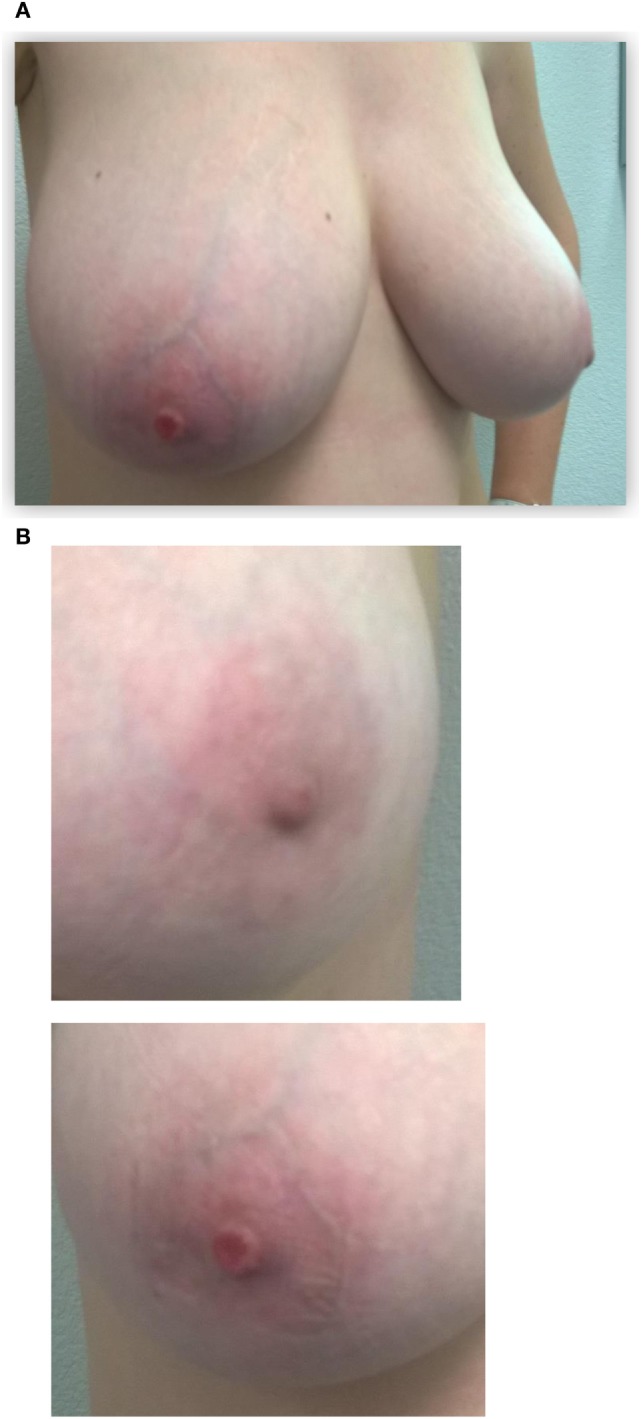
Initial presentation of the patient: **(A)** the right nipple lesion. Initial presentation of the patient: **(B)** macroscopic right nipple lesion, erosion, and redness of the nipple were noted.

**Figure 2:**
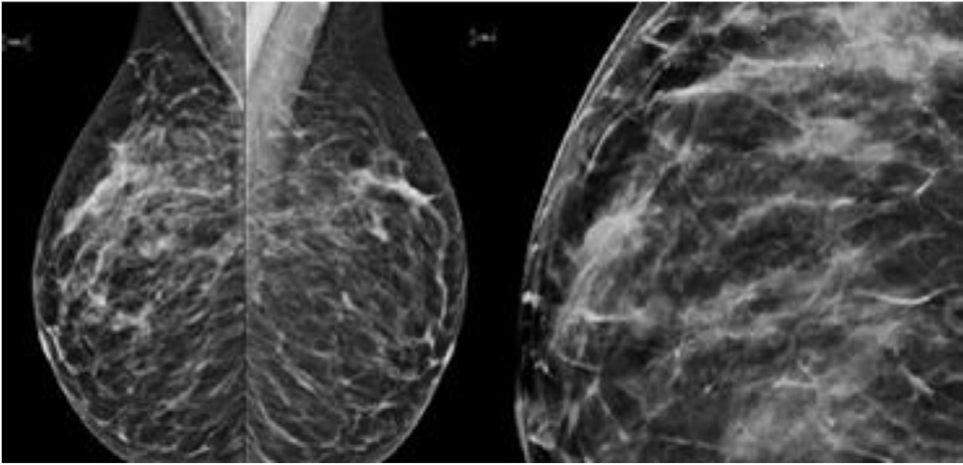
Mammography showed a dense ovoid opacity of 14 mm with irregular suspicious micro calcifications.

**Figure 3:**
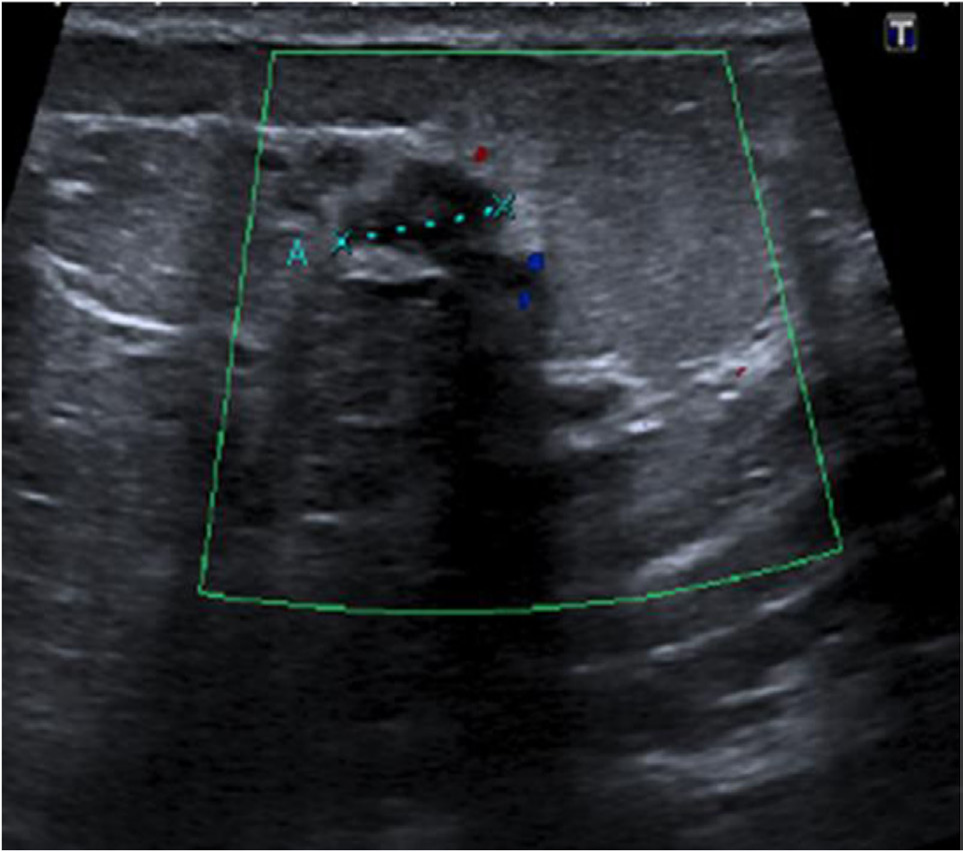
Ultrasound right nipple: suspect galactophoric dilatation of the right supero-external quadrant.

**Figure 4:**
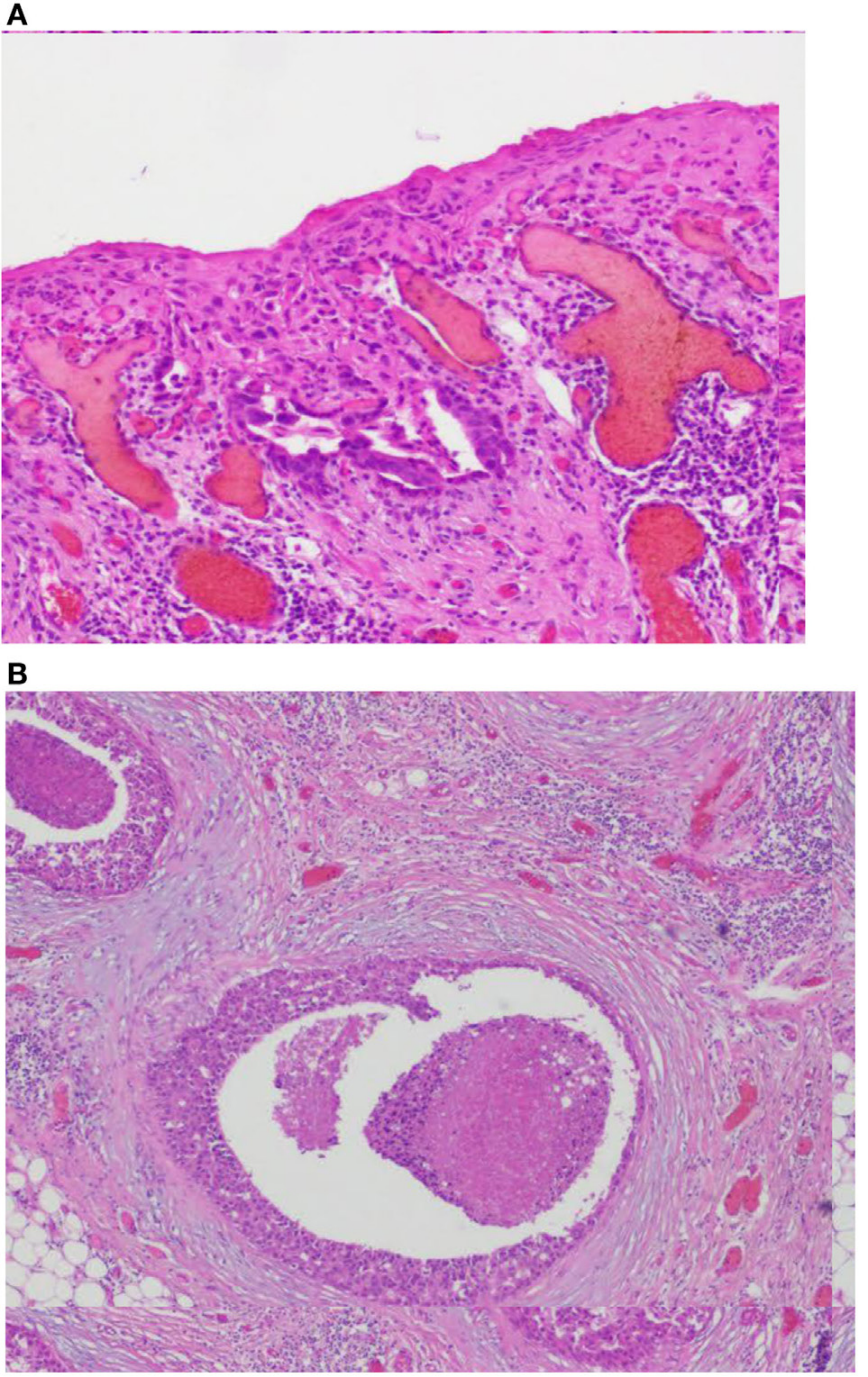
**(A,B)** Images coupe de biopsie. Biopsy concluded to a ductal multicentric carcinoma *in situ*, nuclear grade 2–3, HER2 (+), ER, and PRG (−), classification as B5a. Microscopic examination of the specimen using H&E staining. **(A)** The epidermis of the nipple infiltrated by large Paget’s cells with pale abundant cytoplasm (magnification, ×100). **(B)** Single groups of Paget’s cells with vesicular nuclei and prominent nucleoli (magnification, ×400).

**Figure 5:**
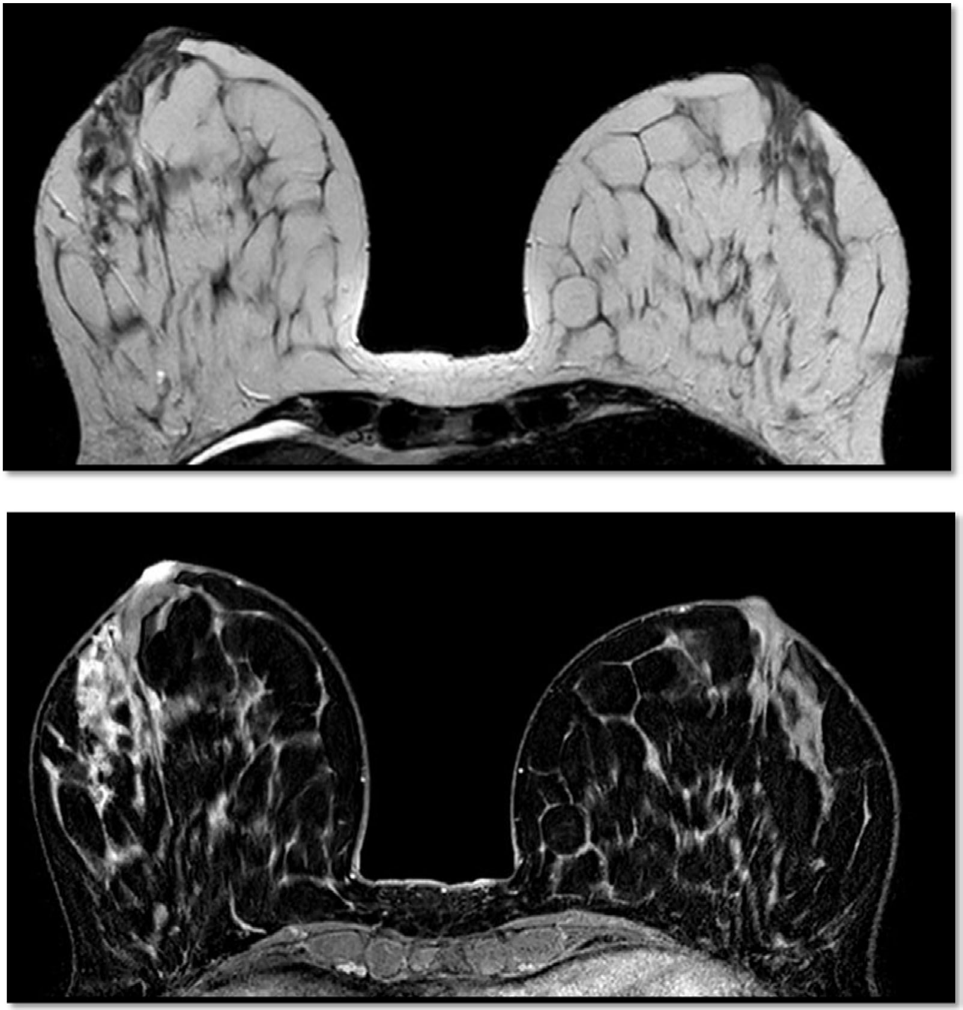
Breast magnetic resonance imaging revealed several multicentric tumoral lesions of the right breast with extension to the nipple–areola complex.

This patient, 43-year-old female (G II PII, height 169 cm, weight 69 kg), under contraception with no family history of cancer, has found a modification of the nipple areolar of the right breast. The examination revealed an eczematous aspect of the right nipple suggesting Paget’s disease (Figures 1A,B). Mammography showed a dense ovoid opacity of 14 mm with irregular suspicious micro calcifications (Figure 2). A suspect galactophoric dilatation of the right supero-external quadrant was seen by ultrasound (Figure 3). Biopsy concluded to a ductal multicentric carcinoma *in situ*, nuclear grade 2–3, HER2 (+), ER, and PRG (−), classification as B5a (Figures 4A,B). Breast magnetic resonance imaging (MRI) revealed several multicentric tumoral lesions of the right breast with extension to the nipple–areola complex (Figure 5). Multidisciplinary meeting proposed a right mastectomy with sentinel lymph node biopsy technique (size of Tumor on IRM). Bilateral radical surgery was performed without complications following the patient wish. Final histology concluded to a high grade DCIS of 40 mm × 35 mm × 25 mm with central foci extending to the main galactophoric ducts associated with a Paget’s disease. Sentinel lymph node was negative.

## Discussion

Paget’s disease was first described by Sir Paget in 1,874 as an eczematous lesion of the nipple associated with an underlying cancer.

Paget’s disease of the breast is a malignant disease that presents itself as eroding and bleeding ulcer of the nipple. It represents an extension of a ductal breast adenocarcinoma. Microscopically, typical large clear cells (Paget’s cells) with pale and abundant cytoplasm and hyperchromatic nuclei with prominent nucleoli are found in the epidermal layer. Paget’s disease is more often associated with primary invasive or *in situ* carcinoma of the breast (1).

## Epidemiology

It’s a rare disease that affects 1–4% of breast cancers. In Switzerland, the incidence rate of breast cancer is 5,861/year, so 32.5% of new cases of cancer per year, and represents 1,384 deaths per year, so 18.9% of cancer deaths in women each year (2). Paget’s disease mainly affects postmenopausal women with an average age of 62.6 years. No clinical or epidemiological predisposing factors have been described; it is isolated on 1.4–13.3%. It is found a homolateral breast cancer on 82–100% cases of which on 13.3–52% CIS and 30–60% invasive (3, 4). *In situ* ductal carcinoma is described multifocal on 42–63% (3).

Paget’s disease of the nipple develops insidiously. Most often unilateral, it initially touches the nipple and then shows a centrifugal growth to reach the areola and then the adjacent skin. It takes the aspect of an eczema sometimes associated with an erythema oozing. The color of the skin changes from pink to red. Retraction, ulceration, or bleeding of the nipple is possible in advanced Paget’s disease. The symptoms usually reported are pruritus, burning, tingling, and pain. In 33% of cases, a palpable mass is present at the time of diagnosis. In 54% of cases, there are enlarged homolateral axillary lymph nodes (5). Diagnostic delay has sometimes been delayed due to an initial dermocorticoid therapy.

## Histopathology

Sir Robert Muir first documented intra epidermal extension of malignant ductal cells through lactiferous channels under the epidermis. The current theory maintains that luminal lactiferous ductal epithelial cells give rise to Paget cells, which migrate in a retrograde fashion into the overlying epidermis. Paget cells possess features of glandular cells and demonstrate positivity for *HER2* oncogene similar to the underlying duct carcinoma cells. The exact mechanisms are less understood, but interactions between heregulin-alpha protein produced by nipple epidermal keratinocytes and *HER2* on the tumor cells have been implicated in the chemotaxis. Alternative hypotheses for Paget disease (PD) include origin from epidermal Toker cells (TCs), which have been considered to be the benign counterparts of Paget cells. Support to this theory was lent by Kuan’s study, which reported the same phenotypic apomucins (MUC1, MUC2, and MUC5AC) in PD as in TCs. The immunoprofile and phenotype of the underlying cancer suggests a common source of origin for Paget cells and TCs. However, there are reports that demonstrate chromosomal alterations in Paget cells are distinct from those in the underlying cancer.

A second theory for the PD origin, independent of the underlying cancer, suggests Paget cells transform *in situ* and derive from cells in the terminal lactiferous duct at its junction with the epidermis. This may explain situations in which PD is not associated underlying carcinoma or is anatomically remote from it. Cytokeratin positive cells (CK 7) are identified in 50% of biopsies from nipple skin around the lactiferous duct ostia. Ultra structurally, desmosomal attachments between Paget cells and keratinocytes, and between Paget cells themselves are noted. These findings suggest that Paget cells may be native to the epidermis and lend support to the *in situ* transformation theory (6).

## Imaging

### Mammography

Whenever Paget’s disease is suspected, a mammogram is performed to detect micro calcifications, heterogeneous, poorly limited with suspicious opacities. The sensitivity of the mammography to detect a tumor is 97% in the presence of a palpable mass, whereas it is only 50% in the absence of palpable mass (7).

### Ultrasounds

The mammary echography is executed systematically to find formations that attenuate the ultrasound and to help the biopsy. In a study of 52 cases of breast PD, the ultrasounds found 43 masses and in 35 patients were lobulated or irregularly profiled most (95%) without posterior acoustic shading. The cancer was clinically absent in 10%, to the mammography invisible in 15%, and radiologically (mammography and US) invisible in 13% of the 52 patients (8, 9).

### Magnetic Resonance Imaging

Additional specialized imaging techniques such as MRI may be used to create additional images of the breast and to determine whether an underlying cancer is present (10).

## Treatment

**Figure 6:**
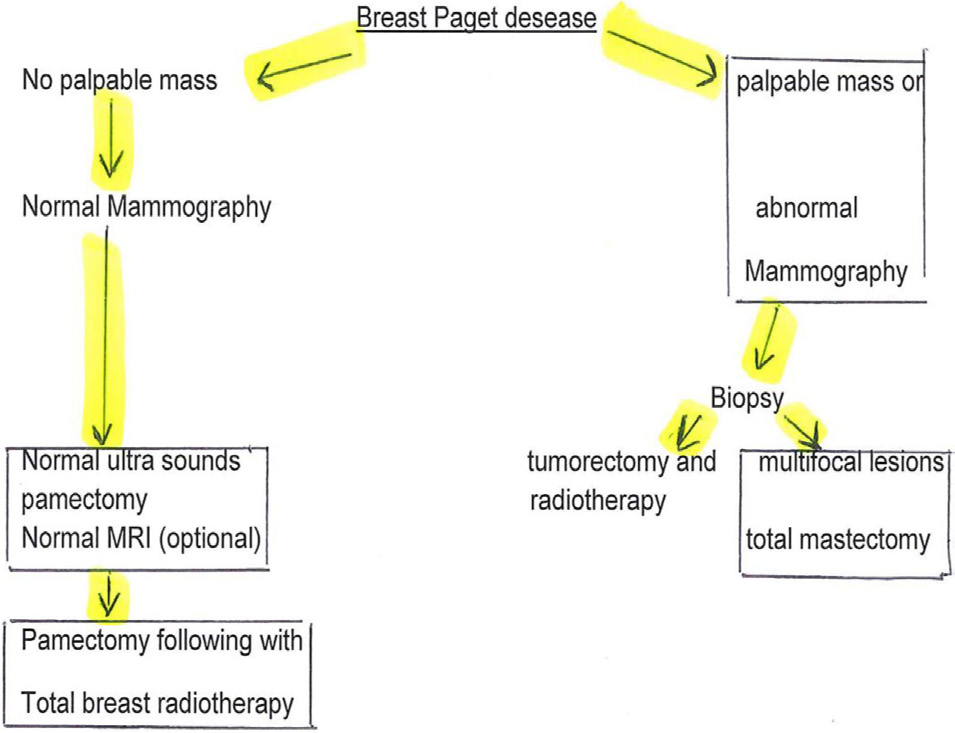
Surgery management’s algorithm of Paget disease.

Mastectomy with or without axillary lymph node dissection has long been regarded as the standard therapy, however, a penectomy with radiotherapy is increasingly chosen. Recent reviews have shown that conservative breast surgery combined with radiation therapy is a feasible alternative for patients with limited disease: long-term breast-conserving surgery would be equivalent to mastectomy in terms of overall disease-free survival. Some authors have suggested this management algorithm (11) (Figure 6).

Currently, the authors recommend increasingly studying the sentinel lymph node in order to avoid the complication of axillary dissection.

## Controversy in the Literature

Beginning in the early 2000s, some authors began comparing the two surgical techniques. A simple mastectomy with or without breast reconstruction is an effective option. In a series of 37 patients treated with simple mastectomy alone, the local recurrence rate was only 5%, with a median follow-up of 40 months (12).

An analysis was carried out of 270 intraductal breast carcinomas in 268 women from 10 institutions in Europe and the United States. In all patients, breast-conserving surgery is followed by radiation therapy. When performed, pathologic axillary lymph node staging was node-negative (*n* = 86). The median follow-up time was 10.3 years (range, 0.9–26.8). The 15-year actuarial overall survival rate was 87%, and the 15-year actuarial cause-specific survival rate was 96%. The 15-year actuarial rate of freedom from distant metastases was 96%. There were 45 local recurrences in the breast, and the 15-year actuarial rate of local failure was 19%. The median time to local failure was 5.2 years (range, 1.4–16.8). A number of clinical and pathological conditions were evaluated for correlation with local failure, and none were predictive for local failure (all *P* ≥ 0.15) (13).

A study of 36 patients with Paget’s disease and no underlying palpable mass or mammographic anomaly, which underwent complete or partial resection of the areola followed by radiotherapy, showed a local recurrence rate of 11% at one follow-up median of 112 months. Of the 22 patients who underwent complete resection of the areola-nipple plaque, followed by total radiotherapy with boost (total dose of 61.5 Gy), three (14%) had local recurrence. In contrast, two of the six women (33%) treated with the same dose of radiotherapy, but only partial resection of the nipple–areola recurrence locally, one had a distant metastasis. The four women with isolated local recurrence were successfully recovered with a mastectomy and remained disease free at the time of the last follow-up (11).

In the prospective study of 61 patients over 75 months, 93% CIS cases, 7% local recurrence in patients with conservative treatment and RTE, so 4 patients including 3 invasive cancers (14).

The prognosis is based on the underlying breast cancer, and treatment should be guided by the stages of the tumor and other prognostic and/or predictive factors (15).

## Conclusion

Paget’s disease of the breast is a rare cancer. It must be discussed in front of an eczematous involvement persistent of the nipple, not responding to a local treatment. There is no evidence at this time that one of the two surgical techniques would improve the survival. The recommendations of the treatment are limited by the absence of randomized prospective trials comparing mastectomy to conservative surgery or by comparing various options for conservative surgery in patients with Paget’s disease of the breast. Most of the reported series are few and patient selection, treatment techniques, and median follow-up vary from study to the other one.

The prognosis depends on the presence of a palpable mass and the invasiveness of the underlying cancer.

We recommend that, depending on the patient’s age, non-conservative surgery should be used if the patient is young in order to limit the risk of local recurrence.

## Ethics Statement

Authors declare that a written informed consent was obtained from the patient for the publication of this case report.

## Author Contributions

All authors have participated to this case.

## Conflict of Interest Statement

The authors declare that the research was conducted in the absence of any commercial or financial relationships that could be construed as a potential conflict of interest.
